# Toxocariasis: a silent threat with a progressive public health impact

**DOI:** 10.1186/s40249-018-0437-0

**Published:** 2018-06-13

**Authors:** Jia Chen, Quan Liu, Guo-Hua Liu, Wen-Bin Zheng, Sung-Jong Hong, Hiromu Sugiyama, Xing-Quan Zhu, Hany M. Elsheikha

**Affiliations:** 1 State Key Laboratory of Veterinary Etiological Biology, Lanzhou Veterinary Research Institute, Chinese Academy of Agricultural Sciences, 1 Xujiaping, Yanchangbu, Lanzhou, Gansu Province 730046 People’s Republic of China; 2 Ningbo University School of Medicine, Ningbo, Zhejiang Province People’s Republic of China; 3 Military Veterinary Institute, Academy of Military Medical Sciences, Key Laboratory of Jilin Province for Zoonosis Prevention and Control, Changchun, Jilin Province People’s Republic of China; 4 College of Veterinary Medicine, Hunan Agricultural University, Changsha, Hunan Province People’s Republic of China; 5 Department of Medical Environmental Biology, Chung-Ang University College of Medicine, Seoul, South Korea; 6 Department of Parasitology, National Institute of Infectious Diseases, Tokyo, Japan; 7 Faculty of Medicine and Health Sciences, School of Veterinary Medicine and Science, University of Nottingham, Loughborough, UK

**Keywords:** *Toxocara canis*, Toxocariasis, Zoonosis, Larva migrans, Epidemiology, Diagnosis, Control

## Abstract

**Background:**

Toxocariasis is a neglected parasitic zoonosis that afflicts millions of the pediatric and adolescent populations worldwide, especially in impoverished communities. This disease is caused by infection with the larvae of *Toxocara canis* and *T. cati*, the most ubiquitous intestinal nematode parasite in dogs and cats, respectively. In this article, recent advances in the epidemiology, clinical presentation, diagnosis and pharmacotherapies that have been used in the treatment of toxocariasis are reviewed.

**Main text:**

Over the past two decades, we have come far in our understanding of the biology and epidemiology of toxocariasis. However, lack of laboratory infrastructure in some countries, lack of uniform case definitions and limited surveillance infrastructure are some of the challenges that hindered the estimation of global disease burden. Toxocariasis encompasses four clinical forms: visceral, ocular, covert and neural. Incorrect or misdiagnosis of any of these disabling conditions can result in severe health consequences and considerable medical care spending. Fortunately, multiple diagnostic modalities are available, which if effectively used together with the administration of appropriate pharmacologic therapies, can minimize any unnecessary patient morbidity.

**Conclusions:**

Although progress has been made in the management of toxocariasis patients, there remains much work to be done. Implementation of new technologies and better understanding of the pathogenesis of toxocariasis can identify new diagnostic biomarkers, which may help in increasing diagnostic accuracy. Also, further clinical research breakthroughs are needed to develop better ways to effectively control and prevent this serious disease.

**Electronic supplementary material:**

The online version of this article (10.1186/s40249-018-0437-0) contains supplementary material, which is available to authorized users.

## Multilingual abstracts

Please see Additional file 1 for translations of the abstract into five official working languages of the United Nations.

## Background

Toxocariasis is a prevalent zoonosis with a significant socioeconomic impact, particularly on impoverished communities around the world. It is caused by nematode parasites of the genus *Toxocara*, of which dog roundworm (*Toxocara canis*; Werner, 1782) and to a lesser extent, cat roundworm (*Toxocara cati*; Schrank, 1788) cause severe disease in humans [1]. In the natural definitive hosts – dogs and cats – these parasitic roundworms colonize the intestinal tract and excrete *Toxocara* eggs with faeces into the environment [2]. The roundworm *Toxocara* is a perfect example of a parasite moving from wild canids to their domestic counterparts and to humans [3, 4]. Dogs or cats, especially in low-income and rural regions, play important roles in the transmission of *Toxocara* spp. through environmental contamination, which spreads the infection to humans [3]. Humans are considered as an accidental or aberrant host, therefore, *Toxocara* larvae cannot develop into adult worms inside the human body [2, 4–6].

Humans acquire infection via ingestion of embryonated/larvated eggs present in the soil or contaminated food, or by ingestion of encapsulated *Toxocara* larvae in improperly cooked tissues of paratenic hosts, such as cows, sheep and chickens [2, 4–9]. Following ingestion of embryonated eggs, larvae hatch in the small intestine, penetrate the intestinal wall, gain access to circulation, migrate throughout the body, leading to a marked inflammatory response and various clinical symptoms, depending on the organ involved [1, 7, 9, 10]. Although infection in humans can be asymptomatic, *Toxocara* parasite has a notorious tendency to cause extra-intestinal pathologies [7, 8]. Indeed, toxocariasis includes four clinical forms, which can lead to serious health consequences [7, 9, 10]. Due to the non-specific symptoms of this disease, its medical and public health impact might be underestimated [11, 12]. Thus, although toxocariasis can be diagnosed tentatively based on patient’s symptoms, laboratory diagnosis is required to improve the accuracy of diagnosis [13, 14].

The first human infection was reported in 1950 [15], and since then it has been reported in almost 100 countries [7, 8]. Over the last few years, toxocariasis has gained an increasing international attention and was listed among the five most neglected parasitic infections according to the US Centers for Disease Control and Prevention (CDC) [3, 4, 12, 16]. The newly sequenced genome of *T. canis* along with transcriptomic analysis has allowed an in-depth characterization of this organism’s molecular characteristics [18]. Also, knowledge of the parasite’s genetic diversity has been improved and new diagnostic markers have been discovered [9, 18–20]. These achievements reflect the increased awareness of toxocariasis and recognition of its continued public health impact. In this article, we provide an updated review of data on toxocariasis, with a focus on the epidemiological, diagnostic and therapeutic aspects of the disease.

## Review

### The causative agents

*Toxocara* spp. are classified under the super-family Ascaridoidea [21–23], and include four valid species, namely *Toxocara canis*, *T. cati*, *T. malaysiensis*, and *T. vitulorum* [21, 22]. Phylogenetic analyses based on the ITS-2 and 28S sequences of the nuclear ribosomal DNA (rDNA), showed that *Toxocara* spp. form a distinct clade, in relation to their definitive hosts, which is separate from *Ascaris* spp. [24]. Moreover, phylogenetic analysis of *T. vitulorum*, *T. canis, T. cati, T. malaysiensis*, *Ascaris suum*, *Anisakis simplex,* and *Onchocerca volvulus* based on amino acid sequences of the entire mitochondrial (mt) genome, revealed that *Toxocara* spp. are more closely related to *A. suum* than to *A. simplex* and *O. volvulus*; and that *T. malaysiensis* is more closely related to *T. cati* than to *T. canis* [19]. *T. vitulorum* has been shown, based on partial mt genome sequence, to be more closely related to *T. malaysiensis* than to *T. canis* and *T. cati* [25]. *T. vitulorum*, *T. canis* and *T. cati*, have been reported worldwide, however *T. malaysiensis* was only reported in China, Malaysia, and Vietnam [26–29].

Neither nucleotide variation of ITS-1 and ITS-2 in *T. canis* from different hosts, including dogs, foxes, and turkey, was observed; nor was there significant intra-specific variability (with none exceeding 0.4%) between specimens from Japan, England, Australia, Sri Lanka and Poland [30]. In regard to *T. cati*, no variation in IT-S sequences within one host was detected, but rDNA microheterogeneity within specimens originating from different geographical locations was reported [28, 31]. Polymorphism analysis of *T. cati* from different geographical locations has shown that differences between Malaysian and Australian strains are 2.9% for ITS-1 and 0.3% for ITS-2 [31], and the ITS sequences of *T. cati* from Poland and Australia differed slightly (0.3–0.4%). However, the differences were much more significant for *T. cati* from Malaysia (2% in ITS-1 and 0.6% in ITS-2) [30]. Interestingly, intra-specific variation in the partial mt sequences within *T. malaysiensis* was only 0.0–0.9% [32]. Microheterogeneity of *T. cati* appears to depend on geographical latitude and it remains to be determined if this heterogeneity plays a role in the response to therapy and potential immune protection.

### Burden of disease by geographic region

Toxocariasis has been reported in many countries worldwide, with most cases occurring in France, Austria, India, Japan, Korea, China, USA, and Brazil (Additional file 2: Table S1). A total of 823 ocular toxocariasis (OT) cases have been reported, including 282 cases in Europe, 317 cases in Asia, five cases in Australia, 218 cases in Latin America, and one documented case in Tunisia (Additional file 2: Table S1). The highest number of OT cases has been reported in Japan and Korea, France, Brazil and the USA. Only 99 neurotoxocariasis (NT) cases have been recorded worldwide, of which 46 cases occurred in Europe, 32 cases in Asia, 20 cases in the Americas, and only one case in South Africa. The largest numbers of NT cases have been reported in Lebanon (17 cases), Sakha Republic in Russian Federation (20 cases) and the USA (8 cases). A total of 247 visceral larva migrans (VLM) cases have been reported worldwide, with the largest numbers reported in Spain (61 cases; 63% of European 97 cases), India (14; 29% of Asian 49 cases), Argentina and Brazil (16; 16% and 76; 75% of cases in South American 101 cases). Eating raw cows’ liver is the main route for acquiring toxocariasis in Japan and Korea, whereas stray dogs and cats spreading eggs in environment are the main source of infection for people in India and other South East Asian countries. In developed countries, including the USA, France and Austria, patients are infected via contact with soil contaminated with *Toxocara* eggs, for example in playgrounds, sandpits and gardens.

Several seroepidemiological features of human toxocariasis are shown in Additional file 2: Table S2. Some risk factors of toxocariasis, such as gender, age, household’s design and construction material, and the presence of domestic animals, have been identified. However, current knowledge of important epidemiological features of toxocariasis, such as the global burden of the disease, disability-adjusted life years, and populations at risk, are still to be determined. Since many infections are asymptomatic and thus can be misdiagnosed, the global burden of toxocariasis is likely to have been underestimated [3]. The global prevalence of human toxocariasis can be influenced by a number of potentially confounding variables that can contribute to differences in the reported toxocariasis prevalence [3, 33]. To improve consistency of the results obtained from prevalence surveys, future studies should consider using standardized diagnostic criteria and should be performed by trained clinicians who can apply a standardized set of toxocariasis case definitions.

### Sources of contamination, route of transmission and reservoirs

Dogs and cats are the most important animal hosts for toxocariasis, especially in developing countries where most cats and dogs have access to public parks and playgrounds, serving as the main source of soil contamination, and posing a huge risk of human exposure to infective eggs (Additional file 2: Table S3). However, in some developed countries, e.g. the UK, urban and rural foxes are the primary source of eggs and infections to humans. Although dogs under six weeks of age excrete more eggs than dogs older than 1 year of age, their lack of access to public areas and the removal of their faeces resulted in ranking foxes as the biggest contributor to eggs (Additional file 2: Table S3). *Toxocara* prevalence is usually higher in cubs, but the prevalence can be high even in adult foxes [34].

Environmental contamination with *Toxocara* eggs is common in most countries, mainly in urban public parks, with positive rates of soil samples obtained from parks ranging from 17.4 to 60.3% in Brazil, 14.4 to 20.6% in the USA, 13.0 to 87.1% in Europe, 30.3 to 54.5% in Africa and 6.6 to 63.3% in Asia [3–44]. In some temperate countries, such as Germany and England, although a few cases of human toxocariasis have been reported [39, 44], environmental contamination with *Toxocara* eggs has been found to be high. The presence of embryonated *Toxocara* eggs attached to the hair of dogs, cats and foxes, represent another route by which humans can acquire infection from dogs or cats [45–48]. Although the total numbers of eggs detected on animal hair varies, puppies and stray animals had higher egg numbers in their coat than others [47, 48].

Evidence suggests that only a handful of animal species might function as paratenic hosts, within which no further development occurs. Paratenic hosts can disseminate infective stages of the parasite and/or aid these stages in avoiding unfavourable conditions during absence of the natural host [6]. These paratenic hosts (animals) include the common shrew (*Sorex araneus*), Eurasian harvest mouse (*Sorex minutes*), Eurasian water shrew (*Neomys fodiens*), Mediterranean water shrew (*Neomy sanomalus*), lesser white-toothed shrew (*Crocidura suaveolens*), common dormouse (*Muscardinus avellanarius*), house mouse (*Mus musculus*), harvest mouse (*Micromys minutus*), striped field mouse (*Apodemus agrarius*), yellow-necked mouse (*Apodemus flavicollis*), wood mouse (*Apodemus silvaticus*), Ural field mouse (*Apodemus microps*), brown rat (*Rattus norvegicus*), bank vole (*Clethrionomys glareolus*), common pine vole (*Pitymys subterraneus*), and common vole (*Microtus arvalis*) [49]. To date, a few studies have identified the prevalence of infection in these animals. In Slovak Republic, 10 non-commensal rodents from suburban locations were confirmed to have higher seropositivity, with the highest seropositivity being found in *Apodemus agrarius* (21%) [50]. In an urban area of Switzerland, four species of non-commensal rodents had a 13.2% *Toxocara* seroprevalence [51].

### Clinical presentation and associated disease syndromes

*Toxocara* infections are often associated with considerable variability in clinical presentation. Because *T. canis* larvae migrate to various body organs, such as the liver, heart, lungs, kidneys, brain, muscle and eyes, a broad range of clinical symptoms can be developed (Table 1) [7]. In general, human toxocariasis is categorized into four clinical forms: VLM, OT, Covert or Common Toxocariasis (CT), and NT, depending on which organs are affected. The severity of disease is dependent on the parasite burden, the duration of larval migration, and age- and immune-mediated responses of the affected individuals [7, 9, 15, 33, 52].

**Table 1:** Characteristics of the different clinical forms of toxocariasis

Clinical syndromes	Population	Involved sites	Associated symptoms
VLM	Children aged 2–7 years	Liver, heart, lungs, kidneys, and muscle	Fever, respiratory symptoms (such as cough, wheeze, dyspnoea, bronchospasm, asthma), hepatomegaly, abdominal pain, vomiting, diarrhoea, anorexia, weight loss, fatigue, neurological manifestations, and pallor [1, 52].
OT	Children aged 5–10 years	Eye	*Toxocara* larval invasion of the peripheral retina and vitreous can cause three major clinical types of OT syndrome over days to weeks: diffuse nematode endophthalmitis, peripheral inflammatory mass type and posterior pole granuloma type [104, 117–119]. Also, diffuse unilateral subacute neuroretinitis (DUSN), bilateral distal symmetric sensory neuropathy (DSN), and choroidal neovascular membrane formation have been attributed to prolonged *Toxocara* infection [1, 120, 121]. Predominantly unilaterally or uncommon bilateral ocular involvement, characterized by visual impairment, strabismus, leukocoria, solid retinal mass predominantly at the posterior pole, vitreous mass or haze, retinal detachment, cataract, endophthalmitis, papillitis, uveitis, as well asvisual loss, vitritis, papillitis and evanescent outer retinal lesions leading to optic atrophy, retinal-artery narrowing and diffuse-pigment epithelial degeneration [1, 122–125].
CT	Children and adults	No specific sites	In adults: breathing difficulties, rash, pruritus, weakness, and abdominal pain, elevated titers of anti-*Toxocara* antibodies, eosinophilia, and elevated total IgE levels [126]. In children: pyrexia, headache, loss of appetite, nausea, emesis, lethargy, behavior and sleep disorders, abdominal pain, pharyngitis, pneumonia, cough, wheeze, itching, rash, limb pains, cervical lymphadenitis, pruritus, rash, and hepatomegaly [52].
NT	Children and adults	Brain and spinal cord	Headache, fever, photophobia, weakness, dorsalgia, confusion, tiredness, visual impairment, epileptic seizures, neuropsychological disturbances, dementia and depression [7, 127–129]. Motor impairment can also be observed in clinical NT cases, such as ataxia, rigor, para- or tetraparesis dysaesthesia, urinary retention, and faecal incontinence [7, 107, 130, 131]. Rarely recognizable neurological signs of eosinophilic meningitis, encephalitis, myelitis, cerebral vasculitis, epilepsy, neuropsychologic deficits or combined pathological presentations, which may be associated with repeated low dose infections, or cerebral vasculitis under anthelmintic therapy, optic neuritis, other cranial nerve involvement, and meningo-radiculitis [6, 7, 127, 132].

*VLM* Visceral larva migrans, *OT* Ocular toxocariasis, *CT* Covert or common toxocariasis, *NT* Neurotoxocariasis, *DUSN* Diffuse unilateral subacute neuroretinitis, *DSN* Distal symmetric sensory neuropathy

#### VLM

VLM is the consequence of a systemic migration of *Toxocara* larvae through the tissue of human viscera. It occurs in children aged 2–7 years and results from high intensity or repeated infections by *T. canis* larvae. Infections in adult individuals have been reported in East Asia (e.g. South Korea and Japan), through ingestion of raw beef, lamb, chicken, or ostrich liver [53, 54]. The liver is the most commonly affected organ in VLM, and is associated with the formation of granulomatous lesions and hepatitis [55–57]. Less frequently, larvae may invade other organs, such as heart, lungs, kidneys and muscle, resulting in myocarditis, myalgia with eosinophilic polymyositis, arthritis and nephritis [58–62]. Dermatological changes, such as rash, pruritus, eczema, panniculitis, urticaria and vasculitis, have also been detected in some VLM cases [63].

#### OT

Common pathologies observed in OT include posterior pole and peripheral retinochoroiditis with granuloma, scleritis, chronic endophthalmitis and panuveitis [7, 8]. Other abnormalities include vitreous opacities, yellowish-white intraretinal lesions in the optic disc with papilledema, live intraocular worm, papillitis, and a tractional retinal detachment, and diffuse unilateral subacute neuroretinitis. The level of visual impairment is dependent on the location of the larvae, the extent of eosinophilia and the fibrotic granulomatous response involved in the induction of distortion, heterotopia and/or detachment of the macula [64, 65].

#### CT

In this form of toxocariasis, patients exhibit non-specific symptoms, such as abdominal pain, fever, anorexia, nausea, headache, vomiting, pharyngitis, pneumonia, cough, wheeze and cervical lymphadenitis, which can be accompanied with eosinophilia and positive *Toxocara* serology [4, 7].

#### NT

NT is caused by invasion of *Toxocara* larvae to the brain and spinal cord, leading to cerebral lesions and neurological damage, predominantly located in the cerebral and cerebellar white matter, with occlusion of cerebral blood vessels (Table 1). The associated clinical symptoms include myelitis, encephalitis, mental confusion and/or meningitis. NT can be influenced by many factors, such as host genetics, the number of ingested ova and prior exposure [8, 66].

### Diagnosis

Misdiagnosis due to the nonspecific clinical presentation, may lead to prolonged morbidity and development of health complications. A high index of suspicion is therefore necessary to establish an early diagnosis and start appropriate treatment. Also, diagnosis of toxocariasis should rely on clinical, radiographic and laboratory evidence of the disease [13, 14]. In general, diagnosis of toxocariasis is based on history (e.g. individuals consumed raw or undercooked meat [7, 13]), clinical examination, direct microscopic examination of tissues (eosinophilic granuloma surrounding live or degenerated roundworm larvae), and blood analysis (leukocytosis and eosinophilia). A range of serological and molecular methods are also available (Table 2) and can be used to confirm the diagnosis.

**Table 2:** Diagnostic methods for toxocariasis

Approaches	Methods	Characteristics	Targets	Advantages	Disadvantages
Direct microscopy [9, 13, 14, 133]	Biopsy and visual detection of the parasite	Invasive, insensitive and time-consuming	Larval sections or eggs	Widely available	Requirement of skilled technicians
Laboratory findings [13, 134, 135]	Blood biochemical analysis	Should be considered in combination with clinical manifestations and further laboratory confirmation	Eosinophilia (average counts of 10 000 cells/mm^3^, approximately 1500 cells/mm^3^in CT, normal range in OT or CT (< 500 cells/mm^3^) or eosinophil cationic protein (ECP) levels (designated as > 28 mg/L)	Useful for detection of active *Toxocara* infections	Non-specific
Antigen detection [13, 14]	Sandwich ELISA	Complex monoclonal antibody (MoAb) production	Circulating TES Ag	Useful for confirmation of active infection	Low sensitivity and specificity
Antibody detection	TES-Ag-ELISA	A standard test for VLM and OT in reference laboratories	Antibodies	Good sensitivity and specificity (70–100%) [4, 13, 14, 33].	Research laboratory use only
	TES-Western blot (24, 28, 30 and 35 kDa fractions of TES Ag)	More specific, but less sensitivity than ELISA [33, 136]		Several commercially available kits in enzyme immunoassay, and Western-blot test formats (ELISA NOVUM, ELISA PU and Toxocara CHEK) [4].	Unavailable for discrimination of past and recent infection
	Recombinant antigens	Less cross-reactivity with antibodies from other helminth infections in endemic regions where poly-parasitism is common, in contrast to TES-Ag [14].	rTES-30, rTES-26 or rTES-120 [67, 136]	Recommended as the best option for diagnosis of human toxocariasis [67, 137–139].	
Nucleic acid amplification [9]	RFLP	Requires a large quantity of genomic DNA, which is not readily available for parasites of small sizes, particularly larvae and eggs	ITS-1 ITS-2	High sensitivity and specificity; useful for species identification and quantification of parasite burden	Technically demanding, requires skilled laboratory technicians
	RAPD	Low reproducibility and specificity; cannot distinguish between eggs of *T. canis* and *T. cati*.			
	PCR	The risk of carry over contamination; low throughput of samples analysis			
	qPCR	Rapid and specific identification of *T. canis* and *T. cati* eggs in faecal and soil samples without the need for additional post-PCR manipulations			
	qPCR	Rapid and specific identification of *T. canis* and *T. cati* eggs in fecal and soil samples without the need for additional post-PCR manipulations			
	LAMP	A cheap, powerful and convenient approach for monitoring the contamination of soil with *Toxocara* eggs			

*OT* Ocular toxocariasis, *CT* Covert or common toxocariasis, *ECP* Eosinophil cationic protein, *VLM* Visceral larva migrans, *TES-Ag Toxocara* excretory secretory antigens

#### Direct microscopy

Demonstrating the presence of *Toxocara* larvae in tissue biopsy, cerebrospinal fluid (CSF) or ocular fluids using direct microscopy remains the “gold standard” for the diagnosis of toxocariasis [13]. However, this method is invasive, insensitive and time-consuming [14]. Also, it can be difficult to distinguish between larvae of *Toxocara* and those of other ascarids, especially when the larvae are degenerated or when only parts of the larva can be recovered from tissues [9, 67].

#### Serodiagnostics

Serological tests are used to support the clinical diagnosis of toxocariasis. Immunoelectrophoresis (IEP) has shown an excellent specificity, but its low sensitivity has limited its utility in clinical settings [13]. Enzyme-linked immunosorbent assay (ELISA), based on excretory and secretory antigens of the third stage larvae (L3) of *T. canis*, is commonly used for diagnosis of human toxocariasis [1]. A limitation of *T. canis* antigen testing is the significant cross-reactivity with other helminths, such as *Ascaris lumbricoides*, particularly in endemic areas [13, 14]. Also, the level of serum IgG can remain elevated for years, which precludes the discrimination between active and persistent infections, especially in patients with high infection intensity [13]. Despite the potential false-positive reaction, these assays have clinical significance that should not be ignored. Although a positive test does not imply causation, a negative test can help to rule out toxocariasis. Serological tests for detecting *T. canis* antibodies may have less value in the evaluation of disease progression in the CNS because results of ELISA for *T. canis* antibodies can be positive in serum, but negative in the CSF of NT patients [68]. Recombinant *Toxocara* antigens have been shown to improve the sensitivity and specificity of serological testing [14]. A combination of diagnostic tests is generally used in seroepidemiological studies (e.g. ELISA is initially used as a fast and relatively inexpensive method, followed by Western blotting to improve the sensitivity and specificity) [13]. Specific detection of total anti-*Toxocara* IgG antibodies and subclasses (e.g. IgG1, IgG2, IgG3 and IgG4) are also possible [69]. Various parasite antigens, semi-purified, and crude antigens from *T. canis* larvae (TCLA), have been used to detect IgM, IgG or IgG4 using ELISA tests with satisfactory sensitivity and specificity [70, 71]. In addition, IgE- and IgM- based ELISAs can be used to evaluate the effect of treatment by monitoring the antibody titer post-treatment [70].

#### Molecular detection

Molecular techniques have high analytical specificity, and shorter turnaround times than other diagnostics. PCR-based assays using a variety of genetic markers (e.g. ITS-1 and ITS-2 regions of rDNA) have been developed and have enabled the identification and phylogenetic analysis of *T. canis*, *T. cati* and other ascarids [72–74]. PCR-based testing has been utilized to identify *T. canis* larvae collected from human biopsies in ocular larva migrans (OT) and from CSF in NT [9, 75, 76]. PCR-based assays, including quantitative real-time PCR (qPCR), PCR-RFLP and PCR-RAPD have been used for accurate identification and diagnosis of *Toxocara* eggs isolated from faeces or soil (Table 2) [2]. The development of loop-mediated isothermal amplification (LAMP) of nucleic acid has provided a rapid and cheap approach for assessing the contamination of soil with *Toxocara* eggs [77, 78]. Molecular methods with improved performance characteristics have the potential to advance the diagnosis of toxocariasis.

#### Diagnostic imaging

A variety of imaging modalities have been used for the detection of lesions caused by infection with *Toxocara* larvae, such as magnetic resonance imaging (MRI), computed tomography (CT), ultrasound, fundus photography, fluorescein angiography, ophthalmic ultrasound and optical coherence tomography (OCT) [79–85].

#### Imaging findings in OT

Fundus photography, fluorescein angiography, ophthalmic ultrasound and OCT can assist in the detection of eye granulomas and in the differentiation of OT from similar ocular conditions, such as retinoblastoma. Routine fundus photography can reveal the location and effects of focal granulomas in eyes with clear media, and in monitoring changes related to disease progression or in response to treatment [86]. Wide-field imaging may aid in the management of patients with peripheral visual involvement. Angiography is used to document the effects of focal and diffuse inflammation on retinal vasculature. Ultrasound biomicroscopy (UBM) can be valuable in detecting the location and extent of vitreous bands and/or traction affecting the anterior segment including the ciliary body, pars plana and peripheral retina [85, 87]. High-penetration optical coherence tomography (HP-OCT) is useful for examining intraretinal lesions, noninvasively. HP-OCT provides clear and continuous scanning from the retina to the choroid [82], compared with conventional OCT.

#### Imaging findings in NT

In NT patients, MRI and CT can be used to detect lesions caused by migrating *Toxocara* larvae in neural tissues. The diagnostic features of NT on MRI include single or multiple, subcortical, cortical or white matter hyperintense lesions on T2-weighted and FLAIR images, and hypointense on T1-weighted images [68, 88]. However, these imaging features are only suggestive, not specific to NT. Therefore, serologic studies of blood and CSF, eosinophilia in the serum or CSF, and clinical and radiological improvement after anthelmintic treatment are necessary to establish the diagnosis.

#### Imaging findings in VLM

VLM lesions appeared on the ultrasound (US) scan as multiple ill-defined, non-spherical hypoechoic lesions [89]. Contrast-enhanced computed tomography of the liver revealed VLM as fluid-attenuating conglomerate lesions [89, 90]. On MRI scan hepatic lesions caused by *Toxocara* L3 larvae migration appeared hypointense on T1-weighted (T1W) images and hyperintense on T2-weighted (T2W) images [89]. VLM lesions exhibited reduced signal intensity on superparamagnetic iron oxide-enhanced T2-weighted MRI images [91]. Based on the radiographic features, fine needle aspiration cytology through the hepatic lesion can be used to characterize the lesion’s content. The presence of mixed inflammatory cells predominantly eosinophils along with Charcot-Leydon crystals in a necrotic background can suggest VLM [90].

#### Differential diagnosis

Despite growing efforts to develop a range of diagnostic methods for detection of human toxocariasis, accurate diagnosis remains a challenge. To improve the management of toxocariasis, we must distinguish this disease from similar conditions. Hence, NT should be differentiated from neural larva migrans (NLM) caused by the nematode *Baylisascaris procyonis*. Also, in clinical cases of meningeal, cerebral, or spinal cord disease with hypereosinophilia of unknown origin and cerebral granulomatous, differential diagnosis of NT should not be overlooked. Differential diagnosis of OT should consider excluding proliferative and neoplastic pathologies (retinoblastoma), and other coexisting parasitic zoonoses (e.g. angiostrongyliasis, toxoplasmosis, cysticercosis, gnathostomiasis, thelaziasis, trichinosis), bacterial infections (e.g. Lyme borreliosis) or viral infections (e.g. cytomegaly). Future research is required to develop better diagnostic methods for detecting the causative parasite, so as to best direct appropriate resources.

## Treatment

The mainstay of toxocariasis therapies includes anthelmintics (e.g. albendazole [ABZ], mebendazole [MBZ] and thiabendazole) and anti-inflammatory drugs [14]. These drugs are used to achieve a clinical resolution or to reduce the damage caused by larval migration to various organs, particularly the brain and eyes [92–94]. ABZ at 400 mg twice a day for five days is the first choice for treatment of VLM patients [1, 13], but MBZ has been indicated as the second therapeutic option for VLM, due to its lower absorption rate outside the gastrointestinal tract compared to ABZ [11, 52]. Other anthelmintic drugs such as diethylcarbamazine (DEC) and ivermectin have been explored to treat VLM, but ivermectin has uncertain efficacy [11, 52]. In cases with cardiac involvement, regimens involving 800 mg/day for two weeks, 50 mg/(kg·day) for 28 days, 600 mg/day for 14 days, or 1000 mg/day for four weeks have been used [58]. Corticosteroids have been used in cases of pulmonary toxocariasis and toxocariasis-associated cardiac diseases [95, 96].

Despite the lack of an optimal treatment for OT, some patients can be treated successfully with anthelmintic drugs or surgically (Table 3), depending on the severity of intraocular inflammation and retinal comorbidities [97–99]. Current standard treatment for OT with active intraocular inflammation includes systemic corticosteroid in combination with ABZ [100]. Periocular or systemic steroids can limit the inflammation, fibrosis, or cicatrization in eyes with active vitritis. Surgery is advised for treatment of structural complications [101–103]. Cryotherapy can be used to treat granulomas, with the administration of steroids following the procedure [104].

**Table 3:** Treatment regimens for human toxocariasis

Clinical forms	Alternatives	Regimens	Remarks	Therapeutic efficacy
VLM				
Albendazole (ABZ) [92, 93, 140]	First choice	400 mg orally twice a day for 5 days	Mild side effects (e.g. dizziness, nausea, abdominal pain) were observed in some patients	The cure rates (45–70%)
Mebendazole (MBZ) [52, 95]	Second choice	100–200 mg orally twice a day for 5 days	Mild side effects similar to adverse effects caused by ABZ	The cure rates (45–70%)
Diethylcarbamazine (DEC) [52, 93]	Alternative choice	40 mg/kg per day for 6 months	Hypersensitivity (e.g. itching, urticaria and edema)	Reduced clinical signs in 70% of patients
Sodium lauryl sulfate containing chitosan-encapsulated ABZ (ABZ/CH); polyethylene glycol (PEG)-conjugated (‘pegylated’) form of ABZ (ABZ/PEG); liposome-encapsulated ABZ stabilized with PEG (ABZ/PEG-LE); phytochemical compounds (compound 17, or C17) [100, 141–144]	Other treatments	Only used in mice models	To increase efficacy, co-administration of a fatty meal with the drugs are recommended for treatment of VLM; liposomal formulations can overcome low drug absorptivity in mice [97, 145]	Uncertain
OT				
Corticosteroid in combination with ABZ [98]	First choice	400 mg of ABZ orally twice a day for 5 days	Prevents scarring, vitreous opacification, membrane formation and vision loss; corticosteroid can increase blood level of ABZ	Uncertain
Surgery [101, 102]	Alternative choice	Vitreoretinal surgery treatment e.g. pars plana vitrectomy (PPV), laser photocoagulation, and cryotherapy	Indicated in cases of retinal detachment, epiretinal membrane, persistent vitreous opacity, and cataracts.	NR
ABZ [99, 101]	Other treatments	200 mg twice a day for one month and 400 or 800 mg twice a day for 2 weeks	Reversible side effects, such as hepatotoxicity, leucopenia, and alopecia; should be avoided during pregnancy	Uncertain
MBZ [99, 101]	Other treatments	20 to 25 mg/kg/day for 3 weeks	The optimal duration of treatment is unknown	Uncertain
Thiabendazole [99, 101]	Other treatments	25 to 50 mg/kg/day for 5–7 days	The optimal duration of treatment is unknown	Uncertain
CT				
ABZ [105]	First choice	200 mg twice a day for one month and 400 or 800 mg twice a day for 2 weeks	ABZ is better tolerated than thiabendazole	Uncertain

*ABZ* Albendazole, *MBZ* Mebendazole, *DEC* Diethylcarbamazine, *NR* Not relevant, *OT* Ocular toxocariasis, *CT* Covert or common toxocariasis, *VLM* Visceral larva migrans

Combinations of corticosteroids with DEC, MBZ, orthiabendazole have been used for the treatment of NT [7]. Although NT may resolve from treatment using ABZ, MBZ, thiabendazole and DEC, ABZ used for at least three weeks, which often needed to be repeated is the preferable choice because it can penetrate the CSF with a minimal toxicity [105, 106]. Corticosteroids can be used for reducing inflammation and controlling hypersensitivity reactions caused by degenerated larvae following the treatment of NT [107]. Encouraging results have been reported recently where a long-term administration of ABZ (10–15 mg/[kg·day]) for four weeks or eight weeks resulted in recovery rate of 78.9 and 81.3%, respectively [11, 108]. Monitoring of side effects post-treatment is recommended especially in patients who might be at a high risk of treatment complications, including people with allergies, pregnant or lactating women, children weighing less than 15 kg, older patients, and those concurrently taking other medicines.

## Prevention

The rapid increase in the number of dogs and cats, especially uncontrolled feral and stray populations and their close proximity to humans, has increased the risk of human infection with *Toxocara* [109]. The lack of an effective method to kill *Toxocara* eggs makes it impossible to eradicate this parasite from the environment [110]. Therefore, strategies for preventing infection should include measures to prevent initial contamination of the environment [2]. Various measures can be implemented to interrupt the transmission of *Toxocara* eggs from animals to humans. These involve de-worming household pets frequently and from a young age. Particular attention and prophylactic anthelmintics should be given to puppies, kittens, or pregnant bitches, which are most likely to transmit the disease. Owners should also safely collect and hygienically dispose of pet faeces, before the eggs become infective. The World Health Organization (WHO) published useful recommendations for disposal of faeces of infected dogs and cats in order to break the dog-soil-human transmission cycle of toxocariasis [111].

Prevention of human infections can also be achieved by washing hands after touching or playing with pets, or following exposure to potentially contaminated sites. Parents should educate children about basic personal hygienic precautions, such as the need for frequent hand washing and the dangers of eating dirt. Children’s play areas should be regularly cleaned and pets kept out of outdoor play areas (e.g. sandboxes) by covering or fencing them off. In addition to measures mentioned above, other interventions have been tested in animal models and may provide alternatives for the prevention of toxocariasis. For example, probiotics (*Enterococcus faecalis* CECT 7121 and *Saccharomyces boulardii*) and DNA-based vaccines (pcDNA3/CpG and pcDNA3/IL-12) have been tested in animal models. *E. faecalis* CECT 7121 and *S. boulardii* significantly reduced the burden of larvae in the liver, lungs and brain significantly [112–114]. DNA vaccination with pcDNA3/CpG and pcDNA3/IL-12 reduced eosinophilia and airway hyper-responsiveness, respectively [115]. Solid lipid nanoparticles of ABZ has been suggested as a promising formulation for the treatment of *T. canis* infection in mice [116].

## Conclusions

Despite extraordinary progress during the past two decades, toxocariasis continues to pose a significant challenge to the public health. This challenge includes a need for continued surveillance to better define the burden of toxocariasis, which requires timely, efficient diagnosis; a need to develop and deploy new drugs and vaccines to combat clinical disease; and a need for ongoing research not only in developing appropriately-targeted prevention strategies, but also in understanding the infection biology of *Toxocara* spp. and human responses to them. Future directions in basic and applied research likely will include: (i) molecular characterization of *Toxocara* isolates from clinical and environmental sources to identify novel biomarkers for diagnosis and epidemiological surveys; (ii) better understanding of humoral, innate, and cell-mediated immunity to *Toxocara* infection for development of prophylactic or therapeutic vaccines; and (iii) establishment of a database that includes behavioural, climatic, demographic, ecological, and socioeconomic factors, crucial data for prediction of infection risk, and for improving the effectiveness of public health interventions by focusing on populations with the highest probability of benefit. Successful realization of these research priorities can advance the understanding of toxocariasis and promote the development of new interventions to prevent *Toxocara* infection and minimize its impact on society.

## Additional files

Additional file 1: Multilingual abstracts in the five official working languages of the United Nations. (PDF 254 kb)

Additional file 2: **Table S1.** Number of clinical cases of human toxocariasis by clinical form and country. **Table S2.** Epidemiological characteristics and risk factors for human toxocariasis and references. **Table S3.** Prevalence of Toxocara spp. in definitive hosts by country and animal host species. (PDF 780 kb)

## Abbreviations

ABZ: Albendazole
CT: Computed tomography
CT: Covert or common toxocariasis
DEC: Diethylcarbamazine
FLAIR: Fluid-attenuated inversion recovery
IEP: Immunoelectrophoresis
MBZ: Mebendazole
MRI: Magnetic resonance imaging
NLM: Neural larva migrans
NT: Neurotoxocariasis
OCT: Optical coherence tomography
OLM: Ocular larva migrans
OT: Ocular toxocariasis
TCLA: Crude antigens from *T. canis* larvae
VLM: Visceral larva migrans
WHO: World Health Organization
