# Supplementary material for: Toxocariasis: a silent threat with a progressive public health impact
Source: Infect Dis Poverty. 2018 Jun 13;7:59. doi: 10.1186/s40249-018-0437-0 (PMC5998503; doi:10.1186/s40249-018-0437-0)
Supplement: Supplementary file 1 — Multilingual abstracts in the five official working languages of the United Nations. (PDF 254 kb) [file 40249_2018_437_MOESM1_ESM.pdf]

Translation of the abstract into the five official working languages of the United Nations

داء السهميات: خطرٌ صامت ذو تأثير تدريجي على مستوى الصحة العامة

قدمه: جيا تشن، وتشيوان ليو، وقوه هوا ليو، ووين بن تشنغ، وسونج جونغ هونج، وهيرومو سوجياما، وشينغ كوان تشو وهاني محمد الشيخ

الملخص

المعلومات الأساسية: داء السهميات هو وباء طفيلي الذي يُصيب ملايين الفئات السكانية من الأطفال واليافعين حول العالم، وخاصةً في المجتمعات المحلية الفقيرة . ينجمُ هذا الداء بسبب العدوى باليرقات السهمية الكلبيةو السهمية الهرية، وهي أكثر الديدان الأسطوانية المعوية الطفيلية انتشارًا بين الكلاب والقطط، على التوالي. في هذا المقال، سنعرضُ التطورات الحديثة في مجال علم الأوبئة، والعرض السريري، والتشخيص والمعالجة الدوائية التي أُستخدمت في علاج داء السهميات.

الموضوع الرئيس:على مدى العقدين الماضيين، فقد توصلنا إلى فهم أعمق لداء السهميات من الناحيتين البيولوجية والوبائية . إلا أن، الافتقار إلى البنية الأساسية للمختبرات في بعض البلدان، وعدم وضع تعاريف موحدة للحالات ومحدودية البنية الأساسية للمراقبة هي بعض من التحديات التي تُعيق تقدير العبء الناجم عن الأمراض على الصعيد العالمي. يشمل داء السهميات أربعة أشكال سريرية: حشوي، وبصري، وسري وعصبي. إذ أن التشخيص الخاطئ لأي من هذه الحالات التي تسبب الإعاقة قد يؤدي إلى عواقب صحية وخيمة ويوجبُ نفقات ضخمة على الرعاية الطبية. فمن حسن الحظ أنه تتوفر العديد من نماذج التشخيص، التي في حال أُستخدمت بفعالية بجانب استخدام المعالجة الدوائية المناسبة، من الممكن أن تسبب في خفض أي احتمال غير ضروري لوفاة المريض.

الاستنتاج: بالرغم أنه أُحرز تقدم في معالجة المرضى المصابين بداء السهميات، لا يزال هناك الكثير من العمل لإنجازه في هذا الشأن. يمكن لتنفيذ التقنيات الجديدة والتوصل لفهم أفضل للأسباب المرضية لداء السهميات أن تحدد مؤشرات حيوية تشخيصية جديدة، والتي قد تساعد في رفع مستوى دقة التشخيص. كما أن هناك حاجة إلى المزيد من الإنجازات البحثية السريرية لتطوير طرق أفضل لمكافحة هذا المرض الخطير والوقاية منه بفعالية.

Translated from English version into Arabic by Farah Dar and Bashaier Allam, through

TRANSLATORS
WITHOUT BORDERS

弓首蛔虫病：一种逐渐影响公共卫生的隐形威胁

Jia Chen, Quan Liu, Guo-Hua Liu, Wen-Bin Zheng, Sung-Jong Hong, Hiromu Sugiyama, Xing-Quan Zhu and Hany M. Elsheikha

摘要

引言：弓首蛔虫病是一种被忽视的人兽共患寄生虫病，在全世界范围内流行分布，尤其在贫困地区更为常见。弓首蛔虫病是由犬弓首蛔虫的幼虫和猫弓首蛔虫的幼虫感染人体而引起的。本文对该病的流行病学、临床症状、诊断以及治疗的研究进展进行了概述。

主要内容：在过去的二十年里，在弓首蛔虫病的生物学和流行病学方面取得了重要进展。然而挑战依然存在，例如：一些国家的实验室基础设施缺乏、病例统一界定的缺失以及疾病监控基础设施的不足已成为对该病进行全球疾病负担评价的最大阻碍。弓首蛔虫病有四类临床

类型：内脏、眼、隐密及神经弓首蛔虫病。一旦无法准确诊断这些疾病甚至误诊，均可对患者造成严重的健康威胁以及沉重的医疗负担。幸运的是，已有各种诊断方法可用于诊断弓首蛔虫病，如与恰当的药物治疗有效结合使用，能使患者的病态最小化。
结论：虽然在弓首蛔虫病的研究方面已取得了一定的进展，但还有很多研究工作需要完成。首先，通过应用新技术和更深入解析弓首蛔虫病的致病机制，可鉴定出新的诊断靶标，以利于提高诊断的准确性。其次，临床研究方面需要取得新的突破，研究出更好的方法以有效地控制和预防弓首蛔虫病。

Translated from English version into Chinese by Jia Chen and Xing-Quan Zhu

La toxocarose, menace silencieuse pesant de plus en plus sur la santé publique

Jia Chen, Quan Liu, Guo-Hua Liu, Wen-Bin Zheng, Sung-Jong Hong, Hiromu Sugiyama, Xing-Quan Zhu et Hany M. Elsheikha

Résumé

Contexte : La toxocarose est une zoonose parasitaire négligée qui affecte des millions d'enfants et d'adolescents dans le monde, particulièrement parmi les populations pauvres. Cette maladie est causée par l'infestation par des larves de *Toxocara canis* et de *Toxocara cati*, deux nématodes qui sont les parasites intestinaux le plus répandus des chiens et des chats, respectivement. Cet article passe en revue les récentes avancées dans les domaines de l'épidémiologie, de la clinique, du diagnostic et des pharmacothérapies utilisées pour le traitement de la toxocarose.
Discussion : Bien que notre compréhension de la biologie et de l'épidémiologie de la toxocarose ait beaucoup progressé depuis ces deux dernières décennies, le manque d'infrastructures de laboratoire dans certains pays, le manque de définitions de cas uniformes et les structures de surveillance limitées, entre autres difficultés, ont rendu difficile l'estimation du fardeau mondial de cette maladie. La toxocarose existe sous quatre formes cliniques : viscérale, oculaire, commune (dite occulte ou cachée) et neurologique. Un diagnostic erroné ou la méconnaissance de ces maladies invalidantes, peut entraîner de graves conséquences sanitaires et des frais médicaux considérables. Il existe heureusement de nombreuses modalités de dépistage qui, combinées efficacement avec les traitements médicamenteux appropriés, peuvent réduire au minimum la morbidité évitable chez les patients atteints.
Conclusion : Bien que des progrès dans la prise en charge des patients atteints de la toxocarose aient été réalisés, il reste encore beaucoup à faire. L'utilisation de nouvelles technologies et une meilleure compréhension de la pathogenèse de la toxocarose peuvent permettre l'identification de nouveaux biomarqueurs diagnostiques qui pourraient améliorer la précision du diagnostic. De nouvelles avancées dans les recherches cliniques sont également nécessaires pour mettre au point des moyens plus efficaces de contrôler et d'endiguer cette maladie grave.

Translated from English version into French by Estellaa and Suzanne Assenat, through

Токсокароз: Тихая угроза с растущим влиянием на здоровье населения

Цзя Чен/Jia Chen, Цюань Лю/Quan Liu, Го-Хуа Лю/Guo-Hua Liu, Вэнь-Бинь Чжэнь/Wen-Bin Zheng, Сун-Йон Хун/Sung-Jong Hong, Хирому Сугияма/Hiromu Sugiyama, Син-Цюань Чжу/Xing-Quan Zhu и Хани М.Эльшейка/Hany M. Elsheikha

Основные тезисы:

Справочная информация: Токсокароз представляет собой зоонозное паразитическое заболевание, которому не уделяют должного внимания, но которому подвержены миллионы пациентов детского и взрослого возраста во всем мире, особенно в бедных населенных пунктах. Это заболеваниеразвивается в результате инфекции, вызванной личинками*Токсокара канис*и*Токсокара кати* , наиболее распространенных нематодных паразитов, заражающих кишечник собак и кошек, соответственно В этой статье приведены последние достижения в эпидемиологии, клинических проявлениях, диагностике и фармакотерапии, применяемой для лечениятоксокароза.

Основной текст:На протяжении последних двух десятилетий нам удалось далеко продвинуться в понимании биологии и эпидемиологии токсокароза. Однако, недостаточное оснащение лабораторий в некоторых странах, отсутствие единых определений случая заболеваний и ограниченная надзорная инфраструктура представляют собой проблемы, затрудняющие определение бремени заболевания по всему миру. Токсокароз проявляется в четырех клинических формах: висцеральной, видимой, скрытой и невральной. Ненадлежащее или ошибочное диагностирование любых из этих тяжелых состояний может привести к тяжелым последствиям для здоровья и значительным расходам на медицинское обслуживание. К счастью, имеется множество методов диагностики, которые при эффективном применении наряду с проведением надлежащего фармакологического лечение, могут свести к минимуму любые излишние для пациента клинические проявления.

Вывод:Хотя были достигнуты некоторые успехи в отношении контроля пациентов с токсокарозом, впереди предстоит еще много работы. Внедрение новых технологий и лучшее понимание патогенеза токсокароза может выявлять новые диагностические биомаркёры, которые смогут повысить диагностическую достоверность. Также необходимы дальнейшие открытия в рамках клинических исследований, чтобы разработать лучшие способы эффективного контроля и профилактики данного серьезного заболевания.

Translated from English version into Russian by Helga_Sh and Ekaterina_Rugg, through

Toxocariasis: una amenaza silenciosa con un impacto progresivo en la salud publica

Jia Chen, Quan Liu, Guo-Hua Liu, Wen-Bin Zheng, Sung-Jong Hong, Hiromu Sugiyama, Xing-Quan Zhu y Hany M. Elsheikha

Resumen

Antecedentes: La toxocariasis es una zoonosis parasitaria desatendida que aflige a millones de personas en las poblaciones pediátrica y adolescente de todo el mundo, especialmente en comunidades empobrecidas. Esta enfermedad está causada por la infección por las larvas de *Toxocara canis* y *Toxocara cati*, los parásitos nematodos intestinales más extendidos en perros y gatos, respectivamente. En este artículo, se analizan los avances más recientes en las áreas de epidemiología, presentación clínica, diagnóstico y farmacoterapias que se han utilizado en el tratamiento de la toxocariasis.

Texto principal: En el último par de décadas, hemos llegado lejos en la comprensión de la biología y la epidemiología de la toxocariasis. Sin embargo, la falta de infraestructura en los laboratorios de algunos países, la falta de definiciones de caso uniformes y una infraestructura de vigilancia limitada son algunos de los desafíos que han dificultado la estimación de la carga mundial de morbilidad. La toxocariasis abarca cuatro formas clínicas: visceral, ocular, encubierta y neurológica. El diagnóstico incorrecto de cualquiera de estas enfermedades incapacitantes puede ocasionar graves consecuencias para la salud y un gasto considerable en atención médica. Afortunadamente, se encuentran disponibles múltiples modalidades de diagnóstico que, si se usan de forma efectiva junto con la administración de tratamientos farmacológicos apropiados, pueden minimizar cualquier morbilidad innecesaria del paciente.

Conclusión: Aunque se ha avanzado en el tratamiento de pacientes con toxocariasis, queda mucho trabajo por hacer. La implementación de nuevas tecnologías y un mejor entendimiento de la patogenia de la toxicariasis pueden identificar nuevos marcadores biológicos de diagnóstico, que podrían ayudar a efectuar diagnósticos más precisos. Además, se necesitan más avances en la investigación clínica para desarrollar formas mejores de controlar y prevenir eficazmente esta grave enfermedad.

Translated from English version into Spanish by Ignacio Montalvo and Marc Gonzalez, through

TRANSLATORS
WITHOUT BORDERS
