# Supplementary material for: Toxocariasis: a silent threat with a progressive public health impact
Source: Infect Dis Poverty. 2018 Jun 13;7:59. doi: 10.1186/s40249-018-0437-0 (PMC5998503; doi:10.1186/s40249-018-0437-0)
Supplement: Supplementary file 2 — Table S1. Number of clinical cases of human toxocariasis by clinical form and country. Table S2. Epidemiological characteristics and risk factors for human toxocariasis and references. Table S3. Prevalence of Toxocara spp. in definitive hosts by country and animal host species. (PDF 780 kb) [file 40249_2018_437_MOESM2_ESM.pdf]

Additional file 2: Table S1-S3.

Table S1.

Number of confirmed cases of human toxocariasis by clinical form and country/geographic region

**Country**	**Clinical forms of human toxocariasis ***				
	**VLM**	**OLM**	**NLM**	**CT**	**Total Number**
China [1-4]		46	1		47
Japan [5]	12	105	8		125
Korea [6-9]	8	106	1		115
Iran [10-12]	1	9			10
India [4,13-18]	14	42	2		58
Malaysia [19,20]	1	1			2
Lebanon [21-25]	3	2	17		22
Israel [26-30]	5	1			6
Turkey [25,31-38]	5	3	2	1	11
United States of America [39-44]	9	181	8	1	199
Mexico [25,45-47]		8	6	12	26
Brazil [4,48-58]	76	14	6		96
Argentina [59-61]	16	15		124	155
Chile [62-65]	10	1			11
Canada [66,67]	1	1			2
France [4,25,68-85]	18	70	8	80	176
Austria [4,25]		38	2		40
Belgium [4,25,86,87]	2	22	3		27
United Kingdom [4,25,88-90]	1	57	3	46	106
Germany [4,25,91]		4	6		10
Italy [4,25,92-95]	4	3	2	9	18
Russia [25]		27			27

Australia [25]		5		5
Cambodia [25]		1		1
Sri Lanka [25]		1		1
Sakha Republic [4]			20	20
South Korea [4]			1	1
Caucasia [25]		20		20
Croatia [25]		7		7
Czech [25]		6		6
Denmark [25]		5		5
Greece [4,25]		3	1	4
Hungary [25]		3		3
Northern Ireland [25]		2		2
Poland [4,25]		2	1	3
Poska [25]		1		1
Portugal [25,98]		2		2
Romania [25]		1		1
Serbia [25]		1		1
Slovakia [25]		1		1
Spain [4,25,96,97]	61	1		62
Sweden [25]		1		1
Switzerland [25,99]		2		1
Varaždin, Croatia [25]		1		1
Poland [4,25,100]		2	1	3
Africa [4,25,101]		1	1	2

*An empty space indicates that no data was available on that form of human toxocariasis in the literature.

Table S2:

Seroepidemiological characteristics and risks of human toxocariasis

**Seroepidemiological features of human toxocariasis**	
**Attributes**	**Key points**
Disease of global importance	Although human toxocariasis is not a notifiable disease, it is one of the most frequently encountered helminth infections, and thus is of a major public health impact [4,102,103]. *T. canis* is one of the most prevalent and ubiquitous zoonotic parasite species occurring from the sub-Arctic to the tropics [25,104-107].
Neglected infection of poverty	The higher levels of endemicity have been reported in developing countries, including La Reunion (93%), Marshall Islands (86.8%), Nepal (81%), Indonesia (63.2%), Malaysia (58%), Swaziland (44.6%), Brazil (36%), Nigeria (30%), and China (12.25%) [108-113]. The disease has also been reported in some developed countries, including the USA (13.9%), Japan (30%), Canada (1.7%), New Zealand (0.7%), Australia (7.5%), Poland (15%), Denmark (2.4%), and Ireland (31%) [25,114-118]. In endemic areas, including the United States, *Toxocara* spp. infection is more prevalent in individuals living in poverty, and is one of the important zoonotic infections during childhood [119,120].
More prevalent in rural areas	Seroprevalence is highest in rural areas, ranging from 35% to 42%, falling to 15% to 20% in semirural zones, and down to 2% to 5% in urban areas. These features are presumably attributed to risk factors that increase the opportunities for transmission, such as contact with dogs or cats (untreated and uncontrolled definitive hosts), low immunity, poor hygiene, lower education levels, and poverty [121,122].
Racial preferential exposure	According to the US National Health and Nutrition Examination Survey (NHANES) results, the seroprevalence for toxocariasis was higher in non-Hispanic blacks (21.2%) than in non-Hispanic whites (12%) and Mexican Americans (10.7%), reflecting differential exposure rates linked to ethnic residential clustering and segregation [115,123].
Clustering within households	*Toxocara* seropositivity can be prevalent in certain clusters within households. The household clusters with the highest prevalence were those who had lower socioeconomic status and were less likely to have cats as pets [124].

**Risk factors for human toxocariasis**	
**Variables**	**Key points**
Exposure-related factors	These include cultural, socioeconomic, environmental, and geographic factors [4,25].
Susceptibility-related factors	These include genetic predisposition, age, gender, nutrition, coinfection, and host immunity (innate or acquired immunity) [25,103,125].
Behavior of human and definitive hosts	The behaviors and lifestyle of humans can increase the possibility of exposure to *Toxocara* eggs [25]. Primarily, international migration, urbanization, animal movement can increase the interactions between humans and definitive hosts, leading to the disease becoming an ever-changing public health concern [103].
Companion animals	Companion animals can be potential health hazards of *T. canis* infection. Embryonated *T. canis* eggs have been recovered from the coats of dogs. Hence, leaving pets untreated can result in heavy contamination of the environment with *T. canis* eggs, thus increasing the risk of human infection [126].
Wild animals	Wild animals (e.g. foxes) have increasingly encroached on urban settlements, have been considered as a potential contributor to increasing environmental contamination, especially in Europe [127].

Table S3.

Prevalence of *Toxocara* spp. in definitive hosts by country and animal host species.

Country	Prevalence in different animal host species				
	Dogs	Cats	Stray dogs or cats	Foxes	Soil contamination or other animals
China [128-130]	45.2% (*n*=438; 2007)	17.78% (*n*=360; 2013-2014)	30% (*n*=40; 2012) in stray dogs; 5.1% (*n*=39; 2012) in stray cats	NA	NA
Japan [131-135]	0.2% (*n*=573; 2015)	0.1% (*n*=555; 2011-2012)	25% (*n*=212; 2006-2011) in stray dogs	NA	NA
Korea [136]	0.0 (*n*=245; 2004)	NA	NA	NA	NA
Iran [137-139]	NA	NA	45% (*n*=140; 2012) in stray cats; 29 % (*n*=90; 2011-2012) in stray dogs	NA	10% (*n*=340; 2011-2012) in public parks; 1.68% (*n*=772; 2016) in contaminated vegetables;
India [140-142]	24.3 % dogs (*n*=558; 2014)	NA	31.29 % (*n*=278; 2010-2011) in stray dogs; 59.3% (*n*=27; 2005-2009) in stray cats	NA	4.75% (*n*=105) in public parks
Malaysia [143,144]	NA	NA	9.9% in cats, and 11.9% in dogs (*n*=152; 2013-2014)	NA	95.7% (*n*=300) in playgrounds
Thailand [145-147]	6.6% (*n*=500; 2014)	9.7% (*n*=300; 2014)	22.5% (*n*=200; 1998) in stray dogs	NA	5.71% (*n*=175; 2004) in public areas.
Turkey [148-151]	13.9% (*n*=115; 2006)	62.5% (*n*=6; 2006)		NA	15.05%, (*n*=259;2005-2006) in public parks; 21.56% (*n*=51;2008) in the dog' s hair
Greece [152]	NA	NA	24% (*n*=135; 2017) in stray cats	NA	NA
Brazil [153-158]	0.7% (*n*=3,099; 2005-2014)	2.2% (*n*=502; 2005-2014)	NA	NA	59.4%(*n*=2,520; 2004-2005) in public areas; 58.5 % (*n*=157; 2015) in chicken;29.0% (*n*=1,642; 2013) in sheep; 11.1% (*n*=45; 2006-2007) in wild dogs
USA [159,160]	6.9% (*n*=231;2009)	33% (*n*=263; 2001)	NA	NA	NA
Tunisia [161]	NA	NA	NA	NA	16% (*n*=31; 2008-2011) in jackals
Denmark [162,163]	NA	84.8% (*n*=189; 2014)	NA	60.9% (*n*=384; 2009-2012)	13.1% (*n*=99; 2009-2012) in raccoon dogs
Australia [164-166]	1.2% (*n*=1400; 2008)	3.2% (*n*=1063; 2008)	NA	14.9% (*n*= 147; 2013)	0.5% (*n*=180) in public parks
Slovenia [167]	NA	NA	NA	38.3% (*n*=428; 2002-2005)	NA
Ireland [168]	NA	NA	NA	20% (*n*=91; 2013)	NA
Mexico [169-173]	6.2% (*n*=130; 2003)	42.5% (*n*=520; 2003)	15.1% (*n*=378; 2008) in stray	0.8% (*n*=249; 2003-2004)	NA

			dogs		
Kyrgyzstan [174]	NA	NA	NA	30% (*n*=151; 2006-2007)	NA
Italy [175-178]	6.6% (*n*=502; 2011-2012)	25.2% (*n*=515; 2012-2013)	33.1% (*n*=139; 2013) in stray cats	9.1% (*n*=129; 2004-2006)	43.8 % (*n*=121; 2010) in wildcats
Switzerland [179]	NA	NA	NA	44.3% (*n*=228;1998-2002)	NA
Great Britain [180,181]	2% (2012)	NA	25% (2012) in stray dogs	61.6% (*n*=588;1999-2000)	NA
Southern Belarus [182]	NA	NA	NA	25.5% (*n*=1,307;1981-2001)	NA
Spain [183]	NA	NA	NA	0.0 (*n*=201; 23.5%;1995)	NA
France [178]	NA	6.25% (*n*=96; 2012-2013)	NA	NA	NA
Germany [178]	NA	7.2% (*n*=55; 2012-2013)	NA	NA	NA

*Prevalence percentage is followed by the number of examined animals and the year of the study.

Abbreviation: NA, not applicable.

References

1. Liu Y, Zhang Q, Li J, Ji X, Xu Y, Zhao P. Clinical characteristics of pediatric patients with ocular toxocariasis in China. Ophthalmologica. 2016;235:97–105.
2. Zhang HF, Hua HY, Wang W. Pediatric ocular toxocariasis in Jiangsu province, Eastern China. Southeast Asian J Trop Med Public Health. 2015;46:8–14.
3. Zhou M, Chang Q, Gonzales JA, Chen Q, Zhang Y, Huang X, Xu G, Wang W, Jiang R. Clinical characteristics of ocular toxocariasis in Eastern China. Graefes Arch Clin Exp Ophthalmol. 2012;250:1373–8.
4. Fan CK, Holland CV, Loxton K, Barghouth U. Cerebral toxocariasis: silent progression to neurodegenerative disorders?. Clin Microbiol Rev. 2015;28:663–86.
5. Yamamoto N. [Food-borne toxocariasis]. Jpn J Food Mivrobiol. 2014;3:1–12.
6. Kwon JW, Sim Y, Jee D. Association between in intermediate uveitis and toxocariasis in the Korean population. Medicine (Baltimore). 2017;96: e5829.
7. Jee D, Kim KS, Lee WK, Kim W, Jeon S. Clinical features of ocular toxocariasis in adult Korean patients. Ocul Immunol Inflamm. 2016;24:207–16.
8. Lee IH, Kim ST, Oh DK, Kim HJ, Kim KH, Jeon P, Byun HS. MRI findings of spinal visceral larva migrans of *Toxocara canis*. Eur J Radiol. 2010;75:236–40.
9. Choi JH, Cho JW, Lee JH, Lee SW, Kim HJ, Choi KD. Obstructive hydrocephalus due to CNS toxocariasis. J Neurol Sci. 2013;329:59–61.
10. Ghoroobi J, Mohajerzadeh L, Khoddami M, Mirshemirani A, Sadeghian N, Mahdavi A, Hatefi S. Abdominal mass secondary to human toxocariasis. APSP J Case Rep. 2017;8:4.
11. Rahimi M, Oustad M, Ashrafi A. Demographic and clinical features of pediatric uveitis at a tertiary referral center in Iran. Middle East Afr J Ophthalmol. 2016;23:237–40.
12. Zibaei M, Sadjjadi SM, Jahadi-Hosseini SH. *Toxocara cati* larvae in the eye of a child: a case report. Asian Pac J Trop Biomed. 2014;4:S53-5.
13. Sharma R, Singh BB, Gill JP. Larva migrans in India: veterinary and public health perspectives. J Parasit Dis. 2015;39:604–12.
14. Rohilla S, Jain N, Yadav R, Dhaulakhandi DB. Hepatic visceral larva migrans. BMJ Case Rep. 2013;pii: bcr2013009288.
15. Lim JH. Hepatic visceral larva migrans of *Toxocara canis*. Am J Trop Med Hyg. 2010; 82:520–1.
16. Jagannath PM, Venkataramana NK, Rao SA, Naik AL, Shivakumar SK, Saktepar A, Gopalakrishnan R, Shankar SK. Recurrent cerebral larva migrans: A case report and review of literature. J Pediatr Neurosci. 2009;4:36–40.
17. Mirdha BR, Khokar SK. Ocular toxocariasis in a North Indian population. J Trop Pediatr. 2002;48:328–30.
18. Sahu ES, Pal B, Sharma T, Biswas J. Clinical profile, treatment, and visual outcome of ocular *Toxocara* in a tertiary eye cara centre. Ocul Immunol Inflamm 2016 Nov 30:1–7. [Epub ahead of print]
19. Lee HF, Danaraj TJ. Visceral larva migrans in Malaya. Report of a case. Am J Trop Med Hyg. 1972;21:174–7.
20. Azira NM, Zeehaida M. A case report of ocular toxocariasis. Asian Pac J Trop Biomed. 2011;1:164–5.
21. Traboulsi R, Boueiz A, Kanj SS. Catastrophic aortic thrombosis due to *Toxocara* infection. Scand J Infect Dis. 2007;39:283–5.
22. Madu AA, Mayers M. Ocular manifestation of systemic infections. Curr Opin Ophthalmol. 1996;7:85–90.
23. Feldman GJ, Parker HW. Visceral larva migrans associated with the hypereosinophilic syndrome and

the onset of severe asthma. Ann Intern Med. 1992;116:838–40.

24. Mansour AM, Abiad B, Boulos FI, Alameddine R, Maalouf FC, Bu Ghannam A, Hamam RN. Adult ocular toxocariasis mimicking ciliary body malignancy. Case Rep Med. 2014;2014:368907.
25. Fan CK, Liao CW, Cheng YC. Factors affecting disease manifestation of toxocarosis in humans: genetics and environment. Vet Parasitol.2013;193:342–52.
26. Ben-Ami M, Katzuni E, Hochman A, Antonelli J, Koren A. Toxocariasis in Emek Israel. Harefuah. 1990;119:72–3.
27. Wolach B, Sinnreich Z, Uziel Y, Gotesman G, Pomerantz A. Toxocariasis: a diagnostic dilemma. Isr J Med Sci. 1995;31:689–92.
28. Amir J, Harel L, Eidlitz-Markus T, Varsano I. Lymphedema as a presenting sign of toxocariasis. Infection. 1995; 23:389–90.
29. Sayar D, Mazilis A, Kassem E, Klein A. [Toxocariasis as a cause of hypereosinophilia]. Harefuah. 2009;148:14–6, 89.
30. Beiran I, Cochavi O, Miller B. “Silent” ocular toxocariasis. Eur J Ophthalmol.1998;8:195–6.
31. Merdin A, Ogur E, Çiçek Kolak Ç, Avcı Merdin F. A Rare Cause of Hypereosinophilia: A Case Report. Turkiye Parazitol Derg. 2016;40:114–6.
32. Coşkun F, AkıncıE. Hepatic toxocariasis: a rare cause of right upper abdominal pain in the emergency department.Turkiye Parazitol Derg. 2013;37:151–3.
33. Ecevit Ç, Bağ Ö, Vergin C, Öztürk A. Visceral larva migrans presenting with hypereosinophilia. Turkiye Parazitol Derg. 2013;37:58–60.
34. Demirci M, Unlü M, Fidan F, Kaya S. Eosinophilic pneumonia due to toxocariasis: an adult case report. Turkiye Parazitol Derg. 2012;36:258–9.
35. Inan M, Sakru N, Vatansever U, Bilgi S. Visceral larva migrans presenting as acute abdomen in a child. J Pediatr Surg. 2006;41:e7–9.
36. Ural S, Özer B, Gelal F, DirimErdoğan D, Sezak N, Balık R, Demirdal T, Korkmaz M. [Transverse myelitis associated with toxocariasis and the importance of locally produced antibodies for diagnosis]. Mikrobiyol Bul.2016;50:478–83.
37. Erdem Kivrak E, Sıpahi OR, Korkmaz M, Işikgöz Taşbakan M, Pullukçu H, Arda B, Yamazhan T, Ulusoy S. [A rarely seen cause of brain abscess: neurotoxocariasis]. Mikrobiyol Bul. 2014;48:507–11.
38. Karagöz E, Selek MB, Aydın E, Hatipoğlu M, Turhan V, Acar A, Öncül O, Görenek L. Recurrent toxocariasis due to chronic urticaria and successful treatment with prolonged albendazole therapy. Turkiye Parazitol Derg. 2015;39:83–5.
39. Kaplan KJ, Goodman ZD, Ishak KG. Eosinophilic granuloma of the liver: a characteristic lesion with relationship to visceral larva migrans. Am J Surg Pathol. 2001;25:1316–21.
40. Anderson A, Fordham LA, Bula ML, Blatt J. Visceral larval migrans masquerading as metastatic disease in a toddler with Wilms tumor. Pediatr Radiol. 2006;36:265–7.
41. Centers for Disease Control and Prevention (CDC). Ocular toxocariasis--United States, 2009-2010. MMWR Morb Mortal Wkly Rep. 2011;60:734–6.
42. Woodhall D, Starr MC, Montgomery SP, Jones JL, Lum F, Read RW, Moorthy RS. Ocular toxocariasis: epidemiologic, anatomic, and therapeutic variations based on a survey of ophthalmic subspecialists. Ophthalmology. 2012;119:1211–7.
43. Stewart JM, Cubillan LD, Cunningham ET Jr. Prevalence, clinical features, and causes of vision loss among patients with ocular toxocariais. Retina. 2005;25:1005–13.
44. Eberhard ML, Alfano E. Adult *Toxocara cati* infections in U.S. children: report of four cases. Am J Trop Med Hyg. 1998;59:404–6.

45. Alvarado-Esquivel C. *Toxocara* infection in psychiatric inpatients: a case control seroprevalence study. PLoS One. 2013;8:e62606.
46. Alvarado-Esquivel C. Toxocariasis in waste pickers: a case control seroprevalence study. PLoS One. 2013;8:e54897.
47. Romero Núñez C, Mendoza Martínez GD, Yañez Arteaga S, Ponce Macotela M, Bustamante Montes P, Ramírez Durán N. Prevalence and risk factors associated with *Toxocara canis* in children. ScientificWorldJournal. 2013;2013:572089.
48. Schoenardie ER, Scaini CJ, Brod CS, Pepe MS, Villela MM, McBride AJ, Borsuk S, Berne ME. Seroprevalence of *Toxocara* infection in children from southern Brazil. J Parasitol. 2013;99:537–9.
49. Lambertucci JR, Rayes A, Serufo JC, Teixeira DM, Gerspacher-Lara R, Nascimento E, Brasileiro Filho G, Silva AC. Visceral larva migrans and tropical pyomyositis: a case report. Rev Inst Med Trop Sao Paulo. 1998;40:383–5.
50. Barra LA, dos Santos WF, Chieffi PP, Bedaque EA, Salles PS, Capitão CG, Vianna S, Hanna R, Pedretti Júnior L. [Visceral larva migrans: a mixed form of presentation in an adult. The clinical and laboratory aspects]. Rev Soc Bras Med Trop. 1996;29:373–6.
51. Almeida MT, Ribeiro RC, Kauffman WM, Maluf Júnior PT, Brito JL, Cristofani LM, Jacob CA, Odone-Filho V. Toxocariasis simulating hepatic recurrence in a patient with Wilms' tumor. Med Pediatr Oncol. 1994;22:211–5.
52. Carlucci A, Spera F. [Toxocariasis (visceral larva migrans). A report of a case in an adopted child]. Minerva Pediatr. 1992;44:125–7.
53. Kawakami E, Fagundes Neto U, Wehba J, Patrício FR. Visceral larva migrans in childhood: report of 2 cases. Arq Gastroenterol 1984; 21: 83–7.
54. De Souza EC, Raskin E, Castro L, Muralha L, Souza O, Pena R. Migrating behavior of presumed Toxocara presenting as punctate inner choroidopathy, idiopathic choroidal neovascularization, and diffuse unilateral subacute neuroretinitis. Retin Cases Brief Rep. 2012;6:430–4.
55. Morais FB, Maciel AL, Arantes TE, Muccioli C, Allemann N. [Ultrasonographic findings in ocular toxocariasis]. Arq Bras Oftalmol. 2012;75:43–7.
56. Lampariello DA, Primo SA. Ocular toxocariasis: a rare presentation of a posterior pole granuloma with an associated choroidal neovascular membrane. J Am Optom Assoc. 1999;70:245-52.
57. Zajdenweber ME, Moraes RT, Brasil OM. [Pseudotoxocariasis: a case report]. Arq Bras Oftalmol. 2006;69:119–21.
58. Carvalho da Silva FT, Yamamoto JH, Hirata CE, Nakashima Y, Chieffi PP, Olivalves E. Optical coherence tomography of a subretinal granuloma in simultaneous visceral and ocular larva migrans. Retin Cases Brief Rep. 2008;2:316–8.
59. Altcheh J, Nallar M, Conca M, Biancardi M, Freilij H. [Toxocariasis: clinical and laboratory features in 54 patients]. An Pediatr (Barc). 2003;58:425–31.
60. Smith RJ, Caribaux LJ, Reviglio VE, Lenta J, Juarez CP, Luna JD. [Submacular surgery in ocular toxocariasis: a clinic-pathologic correlation]. Rev Fac Cien Med Univ Nac Cordoba. 2003;60:61–6.
61. Martín UO, Machuca PB, Demonte MA, Contini L. [Analysis of children with a presumptive diagnosis of toxocariasis in Santa Fe, Argentina]. Medicina (B Aires). 2008;68:353–7.
62. Strickler A, Vásquez N, Maggi L, Hernández J, Hidalgo X. [Toxocarosis and acalculous acute cholecystitis: Consequence or coincidence?]. Rev Chilena Infectol. 2016;33:346–51.
63. Sapunar J, Fardella P. [Visceral larval migrans (Human toxocariasis) cause of hypereosinophilia and visceral granulomas in adults]. Bol Chil Parasitol. 1999;54:21–4.
64. Rugiero E, Cabrera ME, Ducach G, Noemi I, Viovy A. [Systemic toxocariasis in the adult patient]. Rev

Med Chil. 1995;123:612–6.
65. Sánchez T JE, López G JP, González N M, Villaseca D E, Manieu M D, Roizen B A, Noemí H I, Viovy AA. [Prevalence of ocular lesions in children seropositive to *Toxocara canis*]. Rev Chilena Infectol. 2011;28:431–4.
66. Fanning M, Hill A, Langer HM, Keystone JS. Visceral larva migrans (toxocariasis) in Toronto. Can Med Assoc J. 1981;124:21–6.
67. Morin, JD. Ocular toxocariasis. Can Med Assoc J. 1981;124:1557.
68. Koumar Y, Lechiche C, Sotto A, Lachaud L. Invasive toxocariasis with hepatic lesions. Med Mal Infect. 2017;47:71–2.
69. Jorge D, Strady C, Guy B, Deslée G, Lebargy F, Dury S. [Multiple pulmonary opacities revealing toxocariasis]. Rev Pneumol Clin. 2016;72:273–6.
70. Levy M, Bourrat E, Baudouin V, Guillem C, Peuchmaur M, Deschênes G, Fila M. *Toxocara canis* infection: Unusual trigger of systemic lupus erythematosus. Pediatr Int. 2015;57:785–8.
71. Vallentin B, Carsin A, Dubus JC. Toxocariasis: An unusual cause of pleural effusion. Pediatr Pulmonol. 2015;50: E35–6.
72. de Boysson H, Martin Silva N, Comoz F, Boutemy J, Bienvenu B. Vasculitis secondary to anti-C1q antibodies induced by Toxocariasis. Infection. 2015;43:755–8.
73. Fellrath JM, Magnaval JF. Toxocariasis after slug ingestion characterized by severe neurologic, ocular, and pulmonary involvement. Open Forum Infect Dis. 2014;1:ofu063.
74. Bellanger AP, Runge M, Wendling D, Humbert P. Lumbar myositis associated with *Toxocara* spp. infection. Reumatol Clin. 2014;10:54–5.
75. Raffray L, Le Bail B, Malvy D. Hepatic visceral larva migrans presenting as a pseudotumor. Clin Gastroenterol Hepatol. 2013;11:e 42.
76. Kreitmann L, Collet F, Gouello JP, Lemyze M. Cough syncope and multiple pulmonary nodules. Intern Emerg Med. 2011;6 377–9.
77. Tadié JM, Chaudeurge A, Lerolle N, Audibert J, Novara A, Fagon JY, Guérot E. [Acute respiratory distress syndrome due to *Toxocara cati* infection]. Rev Mal Respir. 2010;27:505–8.
78. Sauvet F, Graffin B, Leyral G, Chemsi M, Paris JF, Carli P. [Pulmonary eosinophilia in France: possible toxocariasis].Presse Med 2005; 34: 1713-4.
79. Kagialis-Girard S, Mialou V, Ffrench M, Dupuis-Girod S, Pages MP, Bertrand Y. Thrombocytosis and toxocariasis: report of two pediatric cases. Pediatr Blood Cancer. 2005;44:190–2.
80. Bachmeyer C, Lamarque G, Morariu R, Molina T, Bourée P, Delmer A. Visceral larva migrans mimicking lymphoma. Chest. 2003;123:1296–7.
81. Hamidou MA, Fradet G, Kadi AM, Robin A, Moreau A, Magnaval JF. Systemic vasculitis with lymphocytic temporal arteritis and *Toxocara canis* infection. Arch Intern Med. 2002;162:1521–4.
82. Dromer C, Constantin A, Amar J, Caulier M, Billey T, Chamontin B, Magnaval JF, Fournié B. [Rheumatologic aspects of toxocariasis (visceral Larva migrans). Apropos of 2 cases]. Rev Rhum Ed Fr. 1993;60:621–4.
83. Glickman LT, Magnaval JF, Domanski LM, Shofer FS, Lauria SS, Gottstein B, Brochier B. Visceral larva migrans in French adults: a new disease syndrome? Am J Epidemiol. 1987; 125: 1019–34.
84. Despreaux R, Fardeau C, Touhami S, Brasnu E, Champion E, Paris L, Touitou V, Bodaghi B, Lehoang P. Ocular toxocariasis: clinical features and long-term Visual outcomes in adult patients. Am J Ophthalmol. 2016;166:162–8.
85. Magnaval JF. Comparative efficacy of diethylcarbamazine and mebendazole for the treatment of human toxocariasis. Parasitology. 1995;110:529–33.
86. De Cock C, Lemaitre J, Deuvaert FE. Löeffler endomyocarditis: a clinical presentation as right

ventricular tumor. J Heart Valve Dis. 1998;7:668–71.
87. Van Laethem JL, Jacobs F, Braude P, Van Gossum A, Deviere J. *Toxocara canis* infection presenting as eosinophilic ascites and gastroenteritis. Dig Dis Sci. 1994;39:1370–2.
88. Gemmell A. Toxocariasis as a cause of multiple pulmonary nodules in a paediatric patient. BMJ Case Rep. 2015;2015.
89. Emmer A, Surov A, Kornhuber ME. Incidental cerebral toxocarosis as confirmed by cerebrospinal fluid cytology. Am J Med. 2015;128:e35–6.
90. Lobovská A, Zácková M. [The ocular form of toxocariasis]. Cesk Oftalmol. 1994;50:186–90.
91. Sick C, Hennerici MG. Expect the unexpected: a case of isolated eosinophilic meningitis in toxocariasis. Case Rep Neurol. 2014;6:259–63.
92. Qualizza R, Megali R, Incorvaia C. Toxocariasis resulting in seeming allergy. Iran J Allergy Asthma Immunol. 2009; 8:161–4.
93. Leone N, Baronio M, Todros L, David E, Brunello F, Artioli S, Rizzetto M. Hepatic involvement in larva migrans of *Toxocara canis*: report of a case with pathological and radiological findings. Dig Liver Dis. 2006;38:511–4.
94. Verallo O, Fragiotta S, Verboschi F, Vingolo EM. Diagnostic aspects and retinal imaging in ocular toxocariasis: a case report from Italy. Case Rep Med. 2012;2012:984512.
95. Saporito L, Scarlata F, Colomba C, Infurnari L, Giordano S, Titone L. Human toxocariasis: a report of nine cases. Acta Paediatr. 2008;97:1301–2.
96. Rodero M, Cuéllar C, Fenoy S, del Aguila C, Chivato T, Mateos JM, Laguna R. ELISA antibody determination in patients with anisakiosis or toxocariosis using affinity chromatography purified antigen. Allergy Asthma Proc. 2006;27:422–8.
97. Perteguer MJ, Cuéllar C, Guillén JL, Aguila C, Fenoy S, Chivato T, Laguna R. Cross-reactivity between Anisakis simplex sensitization and visceral larva migrans by *Toxocara canis*. Acta Trop. 2003;89:85–9.
98. Marques A, Rodrigues A, Belo S, Guedes ME. Unusual panuveitis in a child: toxocariasis associated with ocular myiasis. BMJ Case Rep. 2014;2014.pii:bcr2014204475.
99. Bächli H, Minet JC, Gratzl O. Cerebral toxocariasis: a possible cause of epileptic seizure in children. Childs Nerv Syst. 2004;20:468–72.
100. Antonowicz A, Skrzypczyk P, Kępa B2, Pańczyk-Tomaszewska M. [Ocular toxocariasis in a boy with idiopathic syndrome - a case report]. Pol Merkur Lekarski. 2016;41:192–5.
101. Nicoletti A, Bartoloni A, Sofia V, Mantella A, Nsengiyumva G, Frescaline G, Preux PM. Epilepsy and toxocariasis: a case-control study in Burundi. Epilepsia. 2007;48:894–9.
102. Smith H, Holland C, Taylor M, Magnaval JF, Schantz P, Maizels, R. How common is human toxocariasis? Towards standardizing our knowledge. Trends Parasitol. 2009;25:182–8.
103. Macpherson CN. The epidemiology and public health importance of toxocariasis: a zoonosis of global importance. Int J Parasitol. 2013;43:999–1008.
104. Rubinsky-Elefant G, Hoshino-Shimizu S, Jacob CM, Sanchez MC, Ferreira AW. Potential immunological markers for diagnosis and therapeutic assessment of toxocariasis. Rev Inst Med Trop Sao Paulo. 2011;53:61–5.
105. Pinelli E, Herremans T, Harms MG, Hoek D, Kortbeek LM. *Toxocara* and *Ascaris* seropositivity among patients suspected of visceral and ocular larva migrans in the Netherlands: trends from 1998 to 2009. Eur J Clin Microbiol Infect Dis. 2011;30:873–9.
106. Messier V, Levesque B, Proulx JF, Rochette L, Serhir B, Couillard M, Ward BJ, Libman MD, Dewailly E, Dery S. Seroprevalence of seven zoonotic infections in Nunavik, Quebec (Canada). Zoonoses Public Health. 2012;59:107–17.

107. Jenkins EJ, Castrodale LJ, de Rosemond SJ, Dixon BR, Elmore SA, Gesy KM, Hoberg EP, Polley L, Schurer JM, Simard M, Thompson RCA. Tradition and transition: parasitic zoonoses of people and animals in Alaska, northern Canada, and Greenland. Adv Parasitol. 2013;82:33-204.
108. Liao CW, Sukati H, D'Lamini P, Chou CM, Liu YH, Huang YC, Chung MH, Mtsetfwa JS, Jonato J, Chiu WT, Chang PW, Du WY, Chan HC, Chu TB, Cheng HC, Su WW, Tu CC, Cheng CY, Fan CK. Seroprevalence of *Toxocara canis* infection among children in Swaziland, southern Africa. Ann Trop Med Parasitol. 2010;104:73-80.
109. Rubinsky-Elefant G, Hirata CE, Yamamoto JH, Ferreira MU. Human toxocariasis: diagnosis, worldwide seroprevalences and clinical expression of the systemic and ocular forms. Ann Trop Med Parasitol. 2010;104:3–23.
110. Fu CJ, Chuang TW, Lin HS, Wu CH, Liu YC, Langinlur MK, Lu MY, Hsiao Wesley WW, Fan CK. Seroepidemiology of *Toxocara canis* infection among primary schoolchildren in the capital area of the Republic of the Marshall Islands. BMC Infect Dis. 2014; 14: 261.
111. Cong W, Meng QF, You HL, Zhou N, Dong XY, Dong W, Wang XY, Qian AD, Zhu XQ. Seroprevalence and risk factors of *Toxocara* infection among children in Shandong and Jilin provinces, China. Acta Trop. 2015;152:215–9.
112. Cong W, Zhang XX, Zhou N, Yu CZ, Chen J, Wang XY, Li B, Qian AD, Zhu XQ. Toxocara seroprevalence among clinically healthy individuals, pregnant women and psychiatric patients and associated risk factors in Shandong Province, Eastern China. PLoS Negl Trop Dis. 2014;8:e3082.
113. Luo ZJ, Wang GX, Yang CI, Luo CH, Cheng SW, Liao L. Detection of circulating antigens and antibodies in Toxocara canis infection among children in Chengdu, China. J Parasitol.1999;85:252–6.
114. Won KY, Kruszon-Moran D, Schantz PM, Jones JL. National seroprevalence and risk factors for zoonotic *Toxocara* spp. infection. Am J Trop Med Hyg. 2008;79:552–7.
115. Hotez PJ. Neglected infections of poverty in the United States of America. PLoS Negl Trop Dis. 2008;2:e256.
116. Lim PK, Yamasaki H, Mak JW, Wong SF, Chong CW, Yap IK, Ambu S, Kumarasamy V. Field evaluation of a rapid diagnostic test to detect antibodies in human toxocariasis. Acta Trop. 2015;148:32–7.
117. Goyette S, Cao Z, Libman M, Ndao M, Ward BJ. Seroprevalence of parasitic zoonoses and their relationship with social factors among the Canadian Inuit in Arctic regions. Diagn Microbiol Infect Dis. 2014;78:404–10.
118. Jarosz W, Mizgajska-Wiktor H, Kirwan P, Konarski J, Rychlicki W, Wawrzyniak G. Developmental age, physical fitness and *Toxocara* seroprevalence amongst lower-secondary students living in rural areas contaminated with *Toxocara* eggs. Parasitology. 2010;137:53–63.
119. Hotez P. Neglected parasitic infections and poverty in the United States. PLoS Negl Trop Dis. 2014;8:e3012.
120. Hotez P. Neglected infections of poverty in the United States and their effects on the brain. JAMA Psychiatry. 2014;71:1099–100.
121. Fillaux J, Magnaval JF. Laboratory diagnosis of human toxocariasis. Vet Parasitol. 2013;193:327–36.
122. Magnaval JF, Glickman LT, Dorchies P, Morassin B. Highlights of human toxocariasis. Korean J Parasitol. 2001;39:1–11.
123. Won K, Kruszon-Moran D, Schantz P, Jones J. National seroprevalence and risk factors for zoonotic *Toxocara* infection. Am J Trop Med Hyg. 2008;79:552–7.
124. Rubinsky-Elefant G, da Silva-Nunes M, Malafronte RS, Muniz PT, Ferreira MU. Human toxocariasis in rural Brazilian Amazonia: seroprevalence, risk factors, and spatial distribution. Am J Trop Med

Hyg. 2008 ;79:93–8.
125. Viney ME, Graham AL. Patterns and processes in parasite coinfection. Adv Parasitol. 2013;82:321–69.
126. Paul M, King L, Carlin EP. Zoonoses of people and their pets: a US perspective on significant pet-associated parasitic diseases. Trends Parasitol. 2010;26:153–4.
127. Deplazes P, van Knapen F, Schweiger A, Overgaauw PA. Role of pet dogs and cats in the transmission of helminthic zoonoses in Europe, with a focus on echinococcosis and toxocarosis. Vet Parasitol. 2011;182:41–53.
128. Yang Y, Liang H. Prevalence and risk factors ofintestinal parasites in cats from China. Biomed Res Int. 2015;2015:967238.
129. Dai RS, Li ZY, Li F, Liu DX, Liu W, Liu GH, He SW, Tan MY, Lin RQ, Liu Y, Zhu XQ. Severe infection of adult dogs with helminths in Hunan Province, China poses significant public health concerns. Vet Parasitol. 2009;160:348–50.
130. Fang F, Li J, Huang T, Guillot J, Huang W. Zoonotic helminths parasites in the digestive tract of feral dogs and cats in Guangxi, China. BMC Vet Res. 2015;11:211.
131. Itoh N, Kanai K, Kimura Y, Chikazawa S, Hori Y, Hoshi F. Prevalence of intestinal parasites in breeding kennel dogs in Japan. Parasitol Res. 2015;114:1221–4.
132. Itoh N, Kanai K, Tominaga H, Kawamata J, Kaneshima T, Chikazawa S, Hori Y, Hoshi F, Higuchi S. Giardia and other intestinal parasites in dogs from veterinary clinics in Japan. Parasitol Res. 2011;109:253–6.
133. Itoh N, Kanai K, Hori Y, Hoshi F, Higuchi S. Prevalence of *Giardia intestinalis* and other zoonotic intestinal parasites in private household dogs of the Hachinohe area in Aomori prefecture, Japan in 1997, 2002 and 2007. J Vet Sci. 2009;10:305–8.
134. Kimura A, Morishima Y, Nagahama S, Horikoshi T, Edagawa A, Kawabuchi-Kurata T, Sugiyama H, Yamasaki H. A coprological survey of intestinal helminthes in stray dogs captured in osaka prefecture, Japan. J Vet Med Sci. 2013;75:1409–11.
135. Itoh N, Ikegami H, Takagi M, Ito Y, Kanai K, Chikazawa S, Hori Y, Hoshi F, Higuchi S. Prevalence of intestinal parasites in private-household cats in Japan. J Feline Med Surg. 2012;14:436–9.
136. Kim YH, Huh S. Prevalence of Toxocara canis, Toxascaris leonina and Dirofilaria immitis in dogs in Chuncheon, Korea (2004). Korean J Parasitol.2005;43:65–7.
137. Berenji F, Movahedi Rudy AG, Fata A, Tavassoli M, Mousavi Bazaz M, Salehi Sangani G. Soil Contamination with *Toxocara* Spp. Eggs in Public Parks of Mashhad and Khaf, North East of Iran. Iran J Parasitol. 2015;10:286–9.
138. Rostami A, Ebrahimi M, Mehravar S, Fallah Omrani V, Fallahi S, Behniafar H. Contamination of commonly consumed raw vegetables with soil transmitted helminth eggs in Mazandaran province, northern Iran. Int J Food Microbiol. 2016;225:54–8.
139. Emamapour SR, Borji H, Nagibi A. An epidemiological survey on intestinal helminths of stray dogs in Mashhad, North-east of Iran. J Parasit Dis. 2015;39:266–71.
140. Thomas D, Jeyathilakan N. Detection of *Toxocara* eggs in contaminated soil from various public places of Chennai city and detailed correlation with literature. J Parasit Dis. 2014;38:174–80.
141. Sahu S, Samanta S, Sudhakar NR, Raina OK, Gupta SC, Maurya PS, Pawde AM, Kumar A. Prevalence of canine toxocariasis in Bareilly, Uttar Pradesh, India. J Parasit Dis. 2014;38:111–5.
142. Borthakur SK, Mukharjee SN. Gastrointestinal helminthes in stray cats (*Felis catus*) from Aizawl, Mizoram, India. Southeast Asian J Trop Med Public Health. 2011;42:255–8.
143. Tun S, Ithoi I, Mahmud R, Samsudin NI, Kek Heng C, Ling LY. Detection of helminth eggs and identification of hookworm ppecies in stray cats, dogs and soil from Klang Valley, Malaysia. PLoS

One. 2015;10:e0142231.
144. Mohd Zain SN, Rahman R, Lewis JW. Stray animal and human defecation as sources of soil-transmitted helminth eggs in playgrounds of Peninsular Malaysia. J Helminthol. 2015;89:740–7.
145. Rojekittikhun W, Chaisiri K, Mahittikorn A, Pubampen S, Sa-Nguankiat S, Kusolsuk T, Maipanich W, Udonsom R, Mori H. Gastrointestinal parasites of dogs and cats in a refuge in Nakhon Nayok, Thailand. Southeast Asian J Trop Med Public Health. 2014;45:31–9.
146. Wiwanitkit V, Waenlor W. The frequency rate of *Toxocara* species contamination in soil samples from public yards in a urban area "Payathai", Bangkok, Thailand. Rev Inst Med Trop Sao Paulo. 2004;46:113–4.
147. Rojekittikhun W, Nuamtanong S, Anantaphruti MT, Pubampen S, Maipanich W, Visedsuk K. *Toxocara* and *Gnathostoma* among stray canines in Bangkok. Southeast Asian J Trop Med Public Health. 1998;29:744–7.
148. Avcioglu H, Burgu A. Seasonal prevalence of *Toxocara* ova in soil samples from public parks in Ankara, Turkey. Vector Borne Zoonotic Dis. 2008;8:345–50.
149. Aydenizöz-Ozkayhan M, Yağci BB, Erat S. The investigation of *Toxocara canis* eggs in coats of different dog breeds as a potential transmission route in human toxocariasis. Vet Parasitol. 2008;152:94–100.
150. Yaman M, Ayaz E, Gül A, Muz MN. [Investigation of helminth infections of cats and dogs in the Hatay province]. Turkiye Parazitol Derg. 2006 30:200–4.
151. Orhun R, Ayaz E. [Prevalence of helminths in dogs in the region of Van and their potential public health significance]. Turkiye Parazitol Derg. 2006;30:103–7.
152. Diakou A, Di Cesare A, Accettura PM, Barros L, Iorio R, Paoletti B, Frangipane di Regalbono A, Halos L, Beugnet F, Traversa D. Intestinal parasites and vector-borne pathogens in stray and free-roaming cats living in continental and insular Greece. PLoS Negl Trop Dis. 2017;11:e0005335.
153. Gennari SM, Ferreira JI, Pena HF, Labruna MB, Azevedo SD. Frequency of gastrointestinal parasites in cats seen at the University of São Paulo Veterinary Hospital, Brazil. Rev Bras Parasitol Vet. 2016;25:423–8.
154. Ferreira JI, Pena HF, Azevedo SS, Labruna MB, Gennari SM. Occurrences of gastrointestinal parasites in fecal samples from domestic dogs in São Paulo, SP, Brazil. Rev Bras Parasitol Vet. 2016;25:435–40.
155. Campos-da-Silva DR, da Paz JS, Fortunato VR, Beltrame MA, Valli LC, Pereira FE. Natural infection of free-range chickens with the ascarid nematode *Toxocara* sp. Parasitol Res. 2015;114:4289–93.
156. Rassier GL, Borsuk S, Pappen F, Scaini CJ, Gallina T, Villela MM, da Rosa Farias NA, Benavides MV, Berne ME. *Toxocara* spp. seroprevalence in sheep from southern Brazil. Parasitol Res. 2013;112:3181–6.
157. Rocha S, Pinto RM, Floriano AP, Teixeira LH, Bassili B, Martinez A, Costa SO, Caseiro MM. Environmental analyses of the parasitic profile found in the sandy soil from the Santos municipality beaches, SP, Brazil. Rev Inst Med Trop Sao Paulo. 2011;53:277–81.
158. Santos JL, Magalhães NB, Dos Santos HA, Ribeiro RR, Guimarães MP. Parasites of domestic and wild canids in the region of Serra do Cipó National Park, Brazil. Rev Bras Parasitol Vet. 2012;21:270–7.
159. Savilla TM, Joy JE, May JD, Somerville CC. Prevalence of dog intestinal nematode parasites in south central West Virginia, USA. Vet Parasitol. 2011;178:115–20.
160. Spain CV, Scarlett JM, Wade SE, McDonough P. Prevalence of enteric zoonotic agents in cats less than 1 year old in central New York State. J Vet Intern Med. 2001;15:33–8.
161. Lahmar S, Boufana B, Ben Boubaker S, Landolsi F. Intestinal helminths of golden jackals and red foxes from Tunisia. Vet Parasitol. 2014;204:297–303.

162. Al-Sabi MN, Halasa T, Kapel CM. Infections with cardiopulmonary and intestinal helminths and sarcoptic mange in red foxes from two different localities in Denmark. Acta Parasitol. 2014;59:98–107.
163. Takeuchi-Storm N, Mejer H, Al-Sabi MN, Olsen CS, Thamsborg SM, Enemark HL. Gastrointestinal parasites of cats in Denmark assessed by necropsy and concentration McMaster technique. Vet Parasitol. 2015;214:327–32.
164. Palmer CS, Thompson RC, Traub RJ, Rees R, Robertson ID. National study of the gastrointestinal parasites of dogs and cats in Australia. Vet Parasitol. 2008;151:181–90.
165. Carden SM, Meusemann R, Walker J, Stawell RJ, MacKinnon JR, Smith D, Stawell AM, Hall AJ. *Toxocara canis*: egg presence in Melbourne parks and disease incidence in Victoria. Clin Exp Ophthalmol. 2003;31:143–6.
166. Dybing NA, Fleming PA, Adams PJ. Environmental conditions predict helminth prevalence in red foxes in Western Australia. Int J Parasitol Parasites Wildl. 2013 2:165–72.
167. Vergles Rataj A, Posedi J, Zele D, Vengušt G. Intestinal parasites of the red fox (*Vulpes vulpes*) in Slovenia. Acta Vet Hung. 2013;61:454–62.
168. Stuart P, Golden O, Zintl A, de Waal T, Mulcahy G, McCarthy E, Lawton C. A coprological survey of parasites of wild carnivores in Ireland. Parasitol Res. 2013;112:3587–93.
169. Hernández-Camacho N, Pineda-López R, López-González CA, Jones RW. Nematodes parasites of the gray fox (*Urocyon cinereoargenteus* Schreber, 1775) in the seasonally dry tropical highlands of central Mexico. Parasitol Res. 2011;108:1425–9.
170. Trejo CA, Romero Núñez C, García Contreras Adel C, Mendoza Barrera GE. Soil contamination by *Toxocara* spp. eggs in a university in Mexico City. Rev Bras Parasitol Vet. 2012; 21:298–300.
171. Rodríguez-Vivas RI, Gutierrez-Ruiz E, Bolio-González ME, Ruiz-Piña H, Ortega-Pacheco A, Reyes-Novelo E, Manrique-Saide P, Aranda-Cirerol F, Lugo-Perez JA. An epidemiological study of intestinal parasites of dogs from Yucatan, Mexico, and their risk to public health. Vector Borne Zoonotic Dis. 2011;11:1141–4.
172. Cantó GJ, García MP, García A, Guerrero MJ, Mosqueda J. The prevalence and abundance of helminth parasites in stray dogs from the city of Queretaro in central Mexico. J Helminthol. 2011;85:263–9.
173. Martínez-Barbabosa I, Vázquez Tsuji O, Cabello RR, Cárdenas EM, Chasin OA. The prevalence of *Toxocara cati* in domestic cats in Mexico City. Vet Parasitol. 2003;114:43–9.
174. Ziadinov I, Deplazes P, Mathis A, Mutunova B, Abdykerimov K, Nurgaziev R, Torgerson PR. Frequency distribution of *Echinococcus multilocularis* and other helminths of foxes in Kyrgyzstan. Vet Parasitol. 2010;171:286–92.
175. Magi M, Macchioni F, Dell'omodarme M, Prati MC, Calderini P, Gabrielli S, Iori A, Cancrini G. Endoparasites of red fox (*Vulpes vulpes*) in central Italy. J Wildl Dis. 2009;45:881–5.
176. Napoli E, Anile S, Arrabito C, Scornavacca D, Mazzamuto MV, Gaglio G, Otranto D, Giannetto S, Brianti E. Survey on parasitic infections in wildcat (*Felis silvestris silvestris* Schreber, 1777) by scat collection. Parasitol Res. 2016;115:255–61.
177. Paoletti B, Traversa D, Iorio R, De Berardinis A, Bartolini R, Salini R, Di Cesare A. Zoonotic parasites in feces and fur of stray and private dogs from Italy. Parasitol Res. 2015;114:2135–41.
178. Beugnet F, Bourdeau P, Chalvet-Monfray K, Cozma V, Farkas R, Guillot J, Halos L, Joachim A, Losson B, Miró G, Otranto D, Renaud M, Rinaldi L. Parasites of domestic owned cats in Europe: co-infestations and risk factors. Parasit Vectors. 2014;7:291.
179. Reperant LA, Hegglin D, Fischer C, Kohler L, Weber JM, Deplazes P. Influence of urbanization on the epidemiology of intestinal helminths of the red fox (*Vulpes vulpes*) in Geneva, Switzerland. Parasitol Res. 2007;101:605–11.

180. Smith GC, Gangadharan B, Taylor Z, Laurenson MK, Bradshaw H, Hide G, Hughes JM, Dinkel A, Romig T, Craig PS. Prevalence of zoonotic important parasites in the red fox (*Vulpes vulpes*) in Great Britain. Vet Parasitol. 2003;118:133–42.
181. Morgan ER, Azam D, Pegler K. Quantifying sources of environmental contamination with *Toxocara* spp. eggs. Vet Parasitol. 2013;193:390–7.
182. Shimalov VV, Shimalov VT. Helminth fauna of the red fox (*Vulpes vulpes* Linnaeus, 1758) in southern Belarus. Parasitol Res. 2003;89:77–8.
183. Alvarez MF, Iglesias R, García J, Paniagua E, Sanmartin ML. Intestinal helminths of the red fox (*Vulpes vulpes* L.) in Galicia (Northwest Spain). Wiad Parazytol. 1995;41:429–42.
